# Human Milk output among mothers previously treated for severe acute malnutrition in childhood in Democratic Republic of Congo

**DOI:** 10.1186/s40795-021-00467-7

**Published:** 2021-10-25

**Authors:** Christine Chimanuka Murhima’Alika, Ghislain Maheshe Balemba, Pacifique Mwene-Batu Lyabayungu, Guy Mulinganya Mulume’oderhwa, Grace Munthali, Victor Owino, Albert Mwembwo Tambwe, Michèle Dramaix, Philippe Donnen, Ghislain Bisimwa Balaluka

**Affiliations:** 1 Ecole Régionale de Santé Publique, Université Catholique de Bukavu, Bukavu, Democratic Republic of Congo; 2 Centre de Recherche en Sciences Naturelles de Lwiro, Lwiro, Democratic Republic of Congo; 3 Faculty of Medicine, Hôpital Provincial Général de Référence de Bukavu, Université Catholique de Bukavu, Bukavu, Democratic Republic of Congo; 4 Ecole de Santé Publique, Université Libre de Bruxelles, Brussels, Belgium; 5 National Institute for Scientific and Industrial Research of Zambia (NISIR), Lusaka, Zambia; 6 Nutritional and Health Related Studies Section, Division of Human Health, International Atomic Energy Agency, Vienna, Austria; 7 Ecole de Santé Publique de l’Université de Lubumbashi, Lubumbashi, Democratic Republic of Congo

**Keywords:** Childhood malnutrition, Lactating mother, Body composition, Human milk output, D.R.Congo

## Abstract

**Background:**

Malnutrition is a public health problem, as wasting affects 7.5% of children worldwide. The harmful effects of severe acute malnutrition (SAM) can last a lifetime, but how SAM in childhood affects later breastfeeding ability is not clear. In the present study, we assessed the human milk output and body composition among mothers with a history of childhood SAM.

**Methods:**

This retrospective cohort study was carried out in Miti-Murhesa Health Zone (Democratic Republic of Congo) from January 15 to March 17, 2020. We selected lactating mothers with breastfed infants aged 2–12 months. Two categories of mothers were included: those who had been treated for SAM during their childhood (years 1988–2003; *n* = 39) and a community control with no history of SAM (*n* = 40). The weight, height, and mid-upper arm circumference were measured and body mass index (BMI) calculated as weight/height^2^. Body composition and human milk output were assessed using standard deuterium dilution methods. Student t and chi^2^ tests ware applied to compare two groups.

**Results:**

The mean age ± standard deviation of the mothers was 24.4 ± 5.1 and 26.0 ± 6.1 years for the SAM and control groups, respectively (*p* = 0.186). The age of their infants was 5.4 ± 2.3 months in both groups (*p* = 0.962). In the SAM and control groups, the mean maternal BMI was 23.8 ± 2.3 and 23.6 ± 3.7 kg/m^2^ (*p* = 0.849), mean Fat Mass 27.1% ± 5.0 and 27.1% ± 5.8% (*p* = 0.708), and the mean Fat Free mass 72.9% ± 5.0 and 72.9% ± 5.8% (*p* = 0.998), respectively. Human milk output was 833.7 ± 152.1 g/d in SAM group and 827.4 ± 171.4 g/d in the control group (*p* = 0.864).

**Conclusions:**

We found no significant difference in human milk output and body composition in mothers treated for SAM during childhood compared to community controls.

## Introduction

Malnutrition is a public health problem, especially in low and medium-income countries (LMICs). According to the latest Global Nutrition Report, the prevalence of stunting for children age 6–59 months was 22.2%, or 150.8 million children, in 2018, and approximately 7.5%, or 50.3 million children, suffered from wasting [1, 2]. An estimated 5.8 million deaths occur each year among children under 5 years old [3], 45% of which are attributed to malnutrition [4].

Various studies have shown that malnutrition in childhood has adverse health consequences, including heightened risk of metabolic diseases, such as hypertension, diabetes, and obesity, in adolescence and adulthood [5–8].

Studies conducted on a cohort of former subjects treated for SAM during childhood in the DRC showed that surviving males exposed to childhood SAM had low body weight, short height, but no difference for females [9] . Also subjects exposed to SAM during childhood had an overall higher risk of developing metabolic syndrome, carbohydrate metabolism disorder and a higher prevalence of diabetes and hypertension [10, 11]. SAM survivors also had a low socioeconomic level and reduced cognitive abilities compared to subjects not exposed to SAM [12] .

In addition, the prevalence of malnutrition in the adult population in increasing globally, particularly wasting and obesity [13, 14].

The WHO recommends exclusive breastfeeding in the first 6 months of life, followed by the introduction of nutritious complementary foods and continued breastfeeding for at least 2 years of life to prevent malnutrition among infants and young children [15]. Good breastfeeding practice requires the lactating mother to have an adequate nutritional status, and some studies have established associations between the nutritional parameters of the mother and the weight of the newborn [16–18].

Most authors agree that lactating mothers, excluding those with severe pathologies, have sufficient human milk output to ensure breastfeeding, though there is some variation with, for example, maternal age, parity, the age of the breastfed infant, and the season [19, 20] .

The most common methods of assessing human milk are the daily weighing of the mother-infant pair [21] and the administration of deuterium oxide to the mother [22] and these two methods have just been analyzed recently by Jacqueline K., each of them having its advantages and its limits [23]. Also, a recent study shows the need to understand the milk as a biological system given its complexity and its multiple variations between groups of lactating mothers and for the same lactating mother in the time [24].

Previous studies in Eastern Democratic Republic of Congo (DRC) have shown that, even for lactating mothers with borderline nutritional status, the composition of human milk is adequate, though the daily human milk output may be low in cases of severe undernutrition [25]. At the time, Hennart’s work did not establish a significant difference in the macronutrient content of human milk of malnourished mothers other than a low fat content. However, a recent systematic review shows a positive association between the mother’s body mass index (BMI) and the fat content of human milk, without any difference in the protein and carbohydrate content [26]. So an expert workshop concluded that more studies are needed to better understand the different variations in the quantity and composition of human milk [27].

How exposure to malnutrition in childhood may influence a mother’s the ability to produce adequate breast milk is not clear. Researchers are currently working to highlight the long-term effects of severe acute malnutrition (SAM) in childhood. Body composition analysis and anthropometric measurements provide a better understanding of the double burden of malnutrition [28].

Though the harmful effects of SAM can last a lifetime, how SAM in childhood can affect the ability of mothers to breastfeed their offspring is not yet known.

To the best of our knowledge, no studies have been published on lactation performance and lactating mothers exposed to SAM in childhood who survived in a context of endemic malnutrition without going through the nutritional transition described in industrialized countries [29, 30]. The objective of our study is to assess whether previous exposure to childhood SAM impact human milk production of lactating mothers who have lived in a context of endemic malnutrition. Thus, we sought to compare the Human milk output of lactating mothers exposed to SAM during their childhood to that of lactating mothers who did not suffer from SAM. This should contribute to a better understanding of the long-term effects of SAM in childhood and establish evidence to strengthen breastfeeding practices in a context of endemic malnutrition.

## Materials and methods

### Study setting

The study was carried out in the health zone (HZ) of Miti-Murhesa located 33 km from Bukavu city in the province of South Kivu in Eastern DRC. The HZ central office is located 9 km from the Natural Sciences Research Centre of Lwiro (CRSN) within which the Lwiro Pediatric Hospital (LPH) operates. This HZ is between 1500 and 2000 m above sea level [31]. Rural people live on subsistence farming with traditional food practices in a context of poverty and overcrowding. Breastfeeding is provided for all infants, but weaning occurs early from the first months and is based on unsuitable local foods. The most recent nutritional surveys carried out in the territory of Kabare, which includes four HZs, including Miti-Murhesa, confirm this endemic malnutrition with a constant prevalence of overall acute malnutrition of 11.9% in children aged 6–59 months and a prevalence of chronic malnutrition of 58.4% [32]. National surveys conducted over the past two decades confirm the high prevalence of malnutrition in the province of South Kivu [33].

### Study design and subjects

This was a retrospective cohort study in which we identified lactating mothers who had been exposed to SAM in their childhood and whose infants were aged 2–12 months at the time of enrollment. The mothers had been treated and cured of SAM at LPH during their childhood, between 1988 and 2003. The LPH specialized in the management of malnourished children in the Eastern of The Democratic Republic of Congo. Research is currently underway to study the long-term effects of SAM in adult subjects who were treated for SAM in their childhood between 1988 and 2007. Their clinical and anthropometric parameters were traced through the LPH registers and the electronic database of the LPH [9]. At that time, a diagnosis of SAM at LPH was based on the weight to height ratio plotted on the local child growth curve established by De Meyer in 1959, the presence of nutritional edema, and serum albumin levels [9, 34, 35].

The nutritional status of the women at the time of their admission to the hospital as children was reassessed in relation to the WHO child growth standard of 2006 [36]. A new classification was established according to the following criteria. A child was classed as having SAM if they met at least one of the following criteria: mid-upper arm circumference (MUAC) < 115 mm, weight to height < − 3 Z-score (Z-Sc), and the presence of nutritional edema in the hands and/or feet and/or face. Oedematous malnutrition was defined by the presence of nutritional edema in the hands and/or feet and/or face. Severe wasting was based on a MUAC < 115 mm and/or a weight to height < − 3 Z-Sc without edema. The mixed form was defined by the presence of nutritional edema with either a MUAC < 115 mm or a weight to height < − 3 Z-Sc or both [36]. The latter classification of nutritional status was used for the analyses in this study.

The community controls were not exposed to SAM in their childhood and enrolled from the same villages. Data collection for this study was carried out from January 15 to March 17, 2020.

### Identification of population

Eligible participants were identified by a team of 4 doctors, including the study coordinator, 2 nutritionists, 2 assistant nutritionists, and 16 community health workers (CHWs). Mothers exposed to SAM during childhood were identified from the electronic database and the 1988–2003 LPH registers [35]. By consulting the electronic database and the LPH registers, we categorized lactating mothers if they were previously edematous as SAM-kwashiorkor or SAM-marasmus. The assistant nutritionists and CHWs collaborated with all of the village chiefs of Miti-Murhesa HZ to locate the actual residences of targeted lactating mothers. The community controls were lactating mothers who had children of the same age and were recruited from the same villages as the mothers exposed to SAM considering the closest case according to the door-to-door distance. In recruiting controls lactating mothers, we cross-referenced the records of nutritional centers at the time to exclude all cases that had been admitted to a nutritional center (therapeutic or supplementation). Then the witnesses were confirmed by the CHW and village chiefs as having grown up in well-known families with no history of acute malnutrition. These are lactating mothers who grew up in families known to have a higher socio-economic level and who did not experience food problems in childhood. Both assistant nutritionists and some CHWs have collaborated with the CRSN in a community nutrition program since 1990 and know the majority of children in Miti-Murhesa who have suffered from malnutrition in each village. Lactating mothers identified in different health areas and villages were referred to the four targeted health centers (Buhandahanda, Chegera, Kavumu, Mulungu) for study-related assessments. All mothers and infants were in apparent good health at the time of the study (No clinical signs such as fever, diarrhea, major pain or hospitalization in the previous three months).

### Outcomes

The main expected result is to compare human milk output in the two groups of lactating mothers (exposed to SAM in childhood and unexposed). For this purpose, our study aims to evaluate the quantity of human milk output by the lactating mothers by the deuterium dilution method. And as a secondary objective our study aims to compare the body composition of lactating mothers according to the two-compartment model: Fat Mass (FM) and Fat Free Mass (FFM).

### Sample size

Sample size was determined based on the number of lactating mothers who had been treated for childhood SAM and had infants aged 2–12 months that were identified in the targeted health areas during the study period. Thus we have included a total of 79 mothers, 39 exposed and 40 unexposed. Referring to previous studies whose the minimum size of 27 lactating mothers per group was required based on a mean difference between the two groups of 100 and standard deviation of 130 g/d for 80% power [37–39].; the size of 36 lactating mother per group was associated with a power of 90% if we consider the alpha error of 0.05% [40].

### Anthropometric measurements

For the breast-fed infants, body weight was measured to the nearest 100 g using a Salter scale but no other nutritional assessments were carried out. For lactating mothers, body weight was measured to the nearest 100 g using an electronic scale (OMRON, HN-289-EBK) while the subject was dressed only in light clothing. Height was determined to the nearest 0.1 cm without shoes using a SECA 206 cm® measuring tape attached to a wall. The MUAC was measured using an adult MUAC tape. The anthropometric measurements were carried out in accordance with WHO guidelines [36, 41] and subjected to quality control based on independent measurements by two members of the team. The final measurement was the average of the two. In the case of a deviation of more than 300 g for the weight and 0.5 cm for the height, a third measurement was taken. The average of the two closest measurements was used. Body mass index (BMI) was calculated as weight/height^2^.

### Human milk output and mother’s body composition

The mothers’ body composition and human milk output were determined by the Deuterium oxide Dose-to-Mother Technique (DDMT) using D_2_O in the protocol validated by the IAEA [22, 42]. Doses of D_2_O (99.8 atom % D, Cambridge Isotope Laboratories Inc., Andover, USA) of 30 g were accurately weighed to the nearest 0.001 g in sterile leak-proof autoclavable polypropylene dose bottles on a calibrated analytical balance (Sartorius 0.0001 g; Sartorius AG, Goettingen, Germany) at the Laboratory of the Hôpital Provincial Géneral de Référence Bukavu (University Clinic of Catholic University of Bukavu). After consenting, baseline saliva samples were collected from the mother and baby. The mother was asked to rotate a small ball of cotton wool around their mouth until it was completely soaked with saliva. The cotton ball was placed in a 10-mL sterile disposable syringe and the plunger depressed to transfer the saliva into a 3.6-mL sterile cryovial labeled with the mother’s code, the date, and the time of sample collection. Saliva samples were then collected from the babies by a trained technician using a cotton wool swab wrapped with extra cotton wool. The saliva was collected by moving the swab around the baby’s mouth until it was completely soaked with saliva.

Post-dose saliva samples were collected from the mothers and infants on days 1, 2, 3, 4, 13, and 14 after the 30 g deuterium dose was consumed. Deuterium abundance in the saliva was measured using a Fourier transform infrared spectrometer (Agilent Technologies, Malaysia, model 4500 s), and the enrichment was calculated by subtracting the value of the baseline sample from the value of the post-dose sample [43].

Human milk output was determined based on a two-compartment model described by Coward et al. [44] by fitting the isotopic enrichment data to a model for water turnover in the mothers and infants. Maternal body composition was determined from the mother’s total body water (TBW), which was calculated from they-intercept of the mother’s isotope elimination curve and the weight of D_2_O consumed corrected for non-aqueous isotope exchange. The FFM was determined by dividing TBW by 0.732, the assumed hydration of the FFM [45] . The FM was the difference between body weight and the FFM. Curve fitting and calculation of the output were performed using a spreadsheet template provided by the IAEA.

All methods were carried out in accordance with relevant guidelines and regulations.

### Data analysis

The data were encoded in Microsoft Excel. We used SPSS 23.0 software to analyze the data. For the quantitative variables, we checked the normality with the Normal Plot and the Levene test was applied to check the homoscedacity. The quantitative data were presented as means and standard deviation (SD) and categorical data as numbers and percentages. The student t test was used to compare the body composition, weight, height, and human milk output means. Pearson’s Chi^2^ test was used to compare the proportions (or Fisher’s exact test if the Chi^2^ was not valid). One-way ANOVA was used to compare means in different categories of childhood SAM among mothers in the exposure group. We considered the significance level of 0.05.

## Results

A total of 79 lactating mothers with infants aged 2 to 12 months were included in the study (39 lactating mothers exposed to SAM during childhood and 40 community controls). The characteristics of the mothers and infants are given in Table 1. We found no difference in the weight of breastfed infants or in the weight, height, and MUAC of mothers in the SAM and control groups.

**Table 1:** Characteristics of the mothers and infants

Characteristic	Exposed (*n* = 39)	Community Control (*n* = 40)	*P*-value
**Infants**			
Age, months	5.4 ± 2.3	5.4 ± 2.3	0.96
Female	43.6%	45.0%	0.90
Weight, kg	6.7 ± 1.3	6.6 ± 1.4	0.56
WHZ (mean,SD)	1.04 ± 1.53	0.48 ± 1.80	0.75
% WHZ < −2,0	0.0%	5.0%	0.49*
WAZ (mean,SD)	−0.67 ± 1.2	0.88 ± 1.2	0.43
% WAZ < −2,0	12.8%	15.0%	0.52
HAZ (mean,SD)	−1.89 ± 1.6	- 1.47 ± 2.7	0.42
% HAZ < −2,0	46.2%	37.5%	0.44
**Mothers**			
Age, years	24.4 ± 5.1	26.0 ± 6.1	0.19
Weight, kg	55.2 ± 7.5	55.9 ± 7.0	0.69
Height, cm	152.2 ± 6.3	153.7 ± 5.1	0.24
MUAC, mm	253.8 ± 26.4	258.9 ± 20.0	0.34
MUAC < 230 mm	7.7%	10.0%	1.00*
Height < 150 cm	35.9%	22.5%	0.19
Height < 145 cm	10.3%	2.5%	0.20*
Weight < 50 kg	28.2%	10.0%	0.05*
Weight < 45 kg	2.6%	7.5%	0.61*
**Mother’s childhood status (n = 39)**			
SAM - with edema (%)	64.1%	–	–
SAM - no edema (%)	35.9%	–	–

*Data are given as mean ± SD or the proportion of participants (%) unless otherwise noted. SAM = severe acute malnutrition; MUAC = mid-upper arm circumference*

* Fischer exact test

The mother’s body composition and lactation performance are described in Table 2. We found no significant differences between the two groups of lactating mothers in regards to the BMI, FM, TBW, and breast milk output.

**Table 2:** Human milk output and Body composition

Variable	Exposed to SAM (*n* = 39)	Community Control (*n* = 40)	*P*-value
BMI, kg/m^2^	23.8 ± 2.3	23.6 ± 3.7	0.85
Body fat, kg	15.1 ± 4.0	15.0 ± 5.0	0.92
Fat mass, %	27.1 ± 5.0	27.1 ± 5.8	0.99
Fat-free mass, kg	40.1 ± 5.1	40.5 ± 4.1	0.69
Fat-Free mass, %	72.9 ± 5.0	72.9 ± 5.8	0.99
Total body water, kg	29.3 ± 3.7	29.7 ± 3.0	0.65
Total body water, %	53.4 ± 3.7	53.4 ± 4.3	0.99
Breast milk output, g/d	833.7 ± 152.2	827.4 ± 171.4	0.86

*Data are presented as mean ± SD. SAM = severe acute malnutrition; MUAC = mid-upper arm circumference*

The body composition and lactation performance of the SAM group based on type of malnutrition during childhood are described in Table 3. Applying the WHO classification criteria (weight to height ratio, edema, and MUAC), we found no significant difference between the human milk output of lactating mothers treated for SAM (edema, severe wasting or mixed-type).

**Table 3:** Characteristics, body composition, and Human milk output by type of malnutrition in childhood (WHO classification)

Variable	SAM edema (*n* = 15)	SAM mixed-type (*n* = 10)	SAM severe wasting (*n* = 11)	*P*-value*
**Infants**				
Age, months	5.3 ± 2.4	4.8 ± 2.1	6.7 ± 1.3	0.66
Weight, kg	6.9 ± 1.5	6.5 ± 1.0	6.6 ± 1.2	0.71
**Mothers**				
Age, years	24.5 ± 5.5	26.4 ± 5.7	22.6 ± 3.3	0.27
Weight, kg	58.1 ± 6.7	54.3 ± 9.4	52.0 ± 5.4	0.11
Height, cm	152.8 ± 5.5	152.7 ± 7.6	150.6 ± 6.7	0.66
MUAC, mm	265.1 ± 19.7	247.9 ± 19.4	252.2 ± 15.7	0.06
**Body composition**				
BMI, kg/m^2^	24.8 ± 1.8	23.2 ± 3.0	22.9 ± 1.7	0.07
Body fat, kg	16.7 ± 3.9	13.9 ± 4.8	14.1 ± 3.2	0.15
Body fat, %	28.7 ± 5.2	25.1 ± 5.6	26.9 ± 4.4	0.23
Fat-Free mass, kg	41.4 ± 5.2	40.4 ± 5.8	37.9 ± 3.6	0.22
Fat-Free mass, %	71.3 ± 5.2	74.9 ± 5.6	73.1 ± 4.4	0.23
Total body water, kg	30.3 ± 3.8	29.6 ± 4.2	27.8 ± 2.7	0.22
Total body water, %	52.2 ± 3.8	54.7 ± 4.1	53.5 ± 3.2	0.24
**Human milk output**				
Breast milk output, g/d	881.3 ± 147.7	872.5 ± 156.3	776.7 ± 135.4	0.18

*Data are presented as mean ± SD*

* ANOVA applied to compare the three SAM groups

## Discussion

The purpose of this study was to compare human milk output and body composition in a group of lactating mothers with a history of SAM in childhood to these values in a community control group. Overall, we did not observe a significant difference between the two groups. To the best of our knowledge, no studies have specifically measured the human milk output and body composition of lactating mothers with a history of SAM in sub-Saharan Africa.

Our findings on human milk output are similar to those described in other studies conducted in the region, which range between 650 and 850 g/d [16, 22] with small differences attributable to a difference in the methods used or the age of the breastfed infants [19]. By using the DDMT, Owino et al. measured breast milk output of 678 ± 285.4 g/d for lactating mothers with infants 9–10 months old recruited in the same HZ without considering the mother’s nutritional status in childhood [37]. These values are higher than those reported by Donnen in 1997 (605 g/day) in mothers whose infants were 2–4 months old [46], but it is difficult to compare these results because the DDMT has more precision than the methods used at the time (weighing of mother and infant).

The observed values are also similar to those reported in other countries by researchers using DDMT. Matsiko et al. recently worked on a mixed sample of breastfeeding mothers recruited in a rural health district in Rwanda and Holland and reported 854.5 ± 222.3 g/d with breastfed infants aged 3–7 months. These mothers had body composition values similar to those observed in our study, but they had a higher average height and weight (162.6 cm and 61.5 kg) [47]. Researchers in other countries, including Malawi [48], South Africa [49], and 12 countries across continents [50], have reported similar values, but with small variations related to the age of the breastfed infant. The human milk output values are also similar to those obtained in other countries, including Indonesia, where breastfeeding practices were assessed using the same method (787 ± 149 g/d) but with mothers whose average BMI and FM are 24.0 kg/m^2^ and 33.4%, respectively [51].

The body composition variables were also similar in the two groups of lactating mothers in this study, probably due to the physiological adaptation mechanism of the mother during pregnancy also applying to mothers with a history of SAM in childhood. Widen and Gallagher [16] reported that pregnant women had gained weight, FM, and TBW during pregnancy, with a subsequent decline postpartum, and this weight gain can persist until 27 weeks postpartum [16, 52], corresponding to the average age of the children of lactating mothers in our study. The authors identified a few predictors of this gestational weight gain: initial BMI, parity, and age. Thus, lean women have greater weight gain than women with a high initial BMI, and primiparous women have greater weight gain than multiparous women [15, 52–55].

In the present study, the lactating mothers with a history of SAM had a similar BMI as the control group. This BMI is similar to European lactating mothers of various nationalities (22.7 ± 2.6 kg/m^2^), but they had more robust anthropometric parameters with a mean weight of 61.7 ± 7.7 kg and height of 164.9 ± 9.0 cm [56]. Our results are also similar to those observed in lactating mothers in rural Pakistan (BMI 22.9 ± 6.1 kg/m^2^; human milk output 757 ± 249 g/d) but with a higher percentage of FM (31 ± 13%) (46). In general, the observed BMI and FM are similar to the values observed in most LMICs, such as Malawi, Bangladesh, Brazil, and Mexico. Only Kenya has a lower reported BMI and percent body fat in one of two studies (BMI 18.6 ± 1.0 kg/m^2^; FM 17 ± 6%); the second study reported a higher FM percentage (22 ± 6%) [57].

Mothers with a history of oedema had higher human milk output than those with severe wasting but with no statistically significant difference, probably due to the small sample size (Table 3). It is possible that mothers with a history of severe wasting are more affected by relative malnutrition because they have a large proportion of subjects with height < 150 cm (50.0% vs. 28.0%) and weight < 50 kg (42.8% vs. 20.0%) compared to the other group. A larger sample size may allow us to understand the differences between exposure to edema and exposure to severe wasting knowing that, during childhood, children may develop both forms at different times!

A few studies have reported a large variation in breast milk output in the context of malnutrition. Hennart showed that malnourished lactating mothers may have a human milk output < 400 g/d [25]. Another study in Kenya showed that high food insecurity is associated with a reduction in daily human milk intake [51]. However, the last two studies refer to mothers examined in the context of present malnutrition without referring to malnutrition in childhood! These results from the Lwiro cohort suggest that the amount of human milk produced is likely to be related to the environmental and nutritional context in which lactating mothers live rather than to their nutritional status during their childhood. This theory has already been evoked by other authors to emphasize that the long-term effects of malnutrition are strongly linked to the context in which malnourished survivors evolve [58].

Despite these results, which provide a general overview of the long-term effects of SAM in childhood on the lactating performance of mothers, our study has two major limitations. First, we did not have data on the nutritional status (anthropometric and clinical parameters) of the community control group during their childhood. Therefore, there is no irrefutable evidence that the community control group did not contain mothers who experienced moderate malnutrition during childhood. At that time, the MUAC was not used to screen cases of malnutrition in the region. The protocol was based on the weight to height ratio plotted on the local child growth curve and clinical signs of edema or severe wasting [34, 35]. However, we can ascertain that the lactating mothers in the control group had never been admitted to the nutritional center outside the LPH and that they never had kwashiorkor or severe marasmus. At the time, there was a community-based program to detect SAM in the villages of the Miti-Murhesa HZ. Also, the control group included mothers from families well known to the community relays and village chiefs, and these various cross testimonies confirmed that these were good families with no history of malnutrition. What is reassuring for our study is that these community control groups represent the local population, and that these subjects do not have experience with nutritional transition because they lived in an environment where malnutrition has remained endemic. The second limitation is the size of the sample even if we had included the maximum number of mothers that it was possible to have during the season. Indeed, we observed a standard deviation of 150 and 170 g/day whereas the initial sample size calculation was based on a standard deviation of 130 g/day in both groups. This reduced the expected power of our study. Also this sample size was small and did not allow us to make comparisons between subgroups (edema and wasting), but it paves the way for further studies in this area.

## Conclusion

The results of our study show that, in general, SAM in childhood does not affect the lactating mother’s ability to produce human milk. We found no difference in the human milk output and body composition of lactating mothers treated for SAM during childhood compared to community controls. These results suggest that all mothers need support to breastfeed optimally.

## Data Availability

All data generated or analyzed during this study are included in this published article [and its supplementary information files].

## Abbreviations

IAEA: International Atomic Energy Agency
BMI: Body mass index
CHW: Community health worker
CEMUBAC: Centre Médical de l’Université Libre de Bruxelles pour ses activités de coopération
CRSN: Centre de Recherche en Sciences Naturelles de Lwiro
DDMT: Deuterium dose mother technique
DRC: Democratic Republic of Congo
FFM: Fat-free mass
FM: Fat mass
FTIR: Fourier transform infrared spectroscopy
GWG: Gestational weight gain
HZ: Health zone
LMIC: Low and medium-income countries
LPH: Lwiro Pediatric Hospital
MUAC: Mid-upper arm circumference
RUCF: Raedy-use complementary food
RIPSEC: Renforcement institutionnel pour les politiques de santé basées sur l’Evidence en République Démocratique du Congo
SAM: Severe acute malnutrition
TBW: Total body water
WHO: World Health Organization
